# Spatial Accessibility and Social Inclusion: The Impact of Portugal's Last Health Reform

**DOI:** 10.1029/2018GH000165

**Published:** 2019-11-19

**Authors:** H. S. Lopes, V. Ribeiro, P. C. Remoaldo

**Affiliations:** ^1^ Lab2PT, Department of Geography/ICS University of Minho Guimarães Portugal; ^2^ IdRA—Climatology Group/Department of Geography/FGH University of Barcelona Barcelona Spain; ^3^ CIPAF ESE de Paula Frassinetti Porto Portugal; ^4^ ESE de Paula Frassinetti Porto Portugal

**Keywords:** transport, accessibility, health‐care planning, GIS

## Abstract

Health policies seek to promote access to health care and should provide appropriate geographical accessibility to each demographical functional group. The dispersal demand of health‐care services and the provision for such services at fixed locations contribute to the growth of inequality in their access. Therefore, the optimal distribution of health facilities over the space/area can lead to accessibility improvements and to the mitigation of the social exclusion of the groups considered most vulnerable. Requiring for such, the use of planning practices joined with accessibility measures. However, the capacities of Geographic Information Systems in determining and evaluating spatial accessibility in health system planning have not yet been fully exploited. This paper focuses on health‐care services planning based on accessibility measures grounded on the network analysis. The case study hinges on mainland Portugal. Different scenarios were developed to measure and compare impact on the population's accessibility. It distinguishes itself from other studies of accessibility measures by integrating network data in a spatial accessibility measure: the enhanced two‐step floating catchment area. The convenient location for health‐care facilities can increase the accessibility standards of the population and consequently reduce the economic and social costs incurred. Recently, the Portuguese government implemented a reform that aimed to improve, namely, the access and equity in meeting with the most urgent patients. It envisaged, in terms of equity, the allocation of 89 emergency network points that ensured more than 90% of the population be within 30 min from any one point in the network. Consequently, several emergency services were closed, namely, in rural areas. This reform highlighted the need to improve the quality of the emergency care, accessibility to each care facility, and equity in their access. Hence, accessibility measures become an efficient decision‐making tool, despite its absence in effective practice planning. According to an application of this type of measure, it was possible to verify which levels of accessibility were decreased, including the most disadvantaged people, with a larger time of dislocation of 12 min between 2001 and 2011.

## Key Points

- Accessibility levels show significant differences between urban and rural areas, namely, with the population of the two‐major urban centers (Metropolitan Area of Porto and Metropolitan Area of Lisbon)
- The Portugal's health reform led to reduction in accessibility levels in rural areas
- Health policies can contribute to mitigate the social exclusion through the provision of localized emergency units of proximity

## 1 Introduction

Inequality in accessing basic services has always been associated to underlying human existence, becoming a crucial factor in the progress and innovation of certain communities. At the same time, it can also be a factor of social exclusion of the most vulnerable groups. The elderly group is the most disadvantaged, as shown in a previous study to access hospital services in mainland Portugal (Lopes et al., 2016). The aging of developed societies in recent decades has tended to lead to and to reflect the multiple effects of social exclusion. Optimal health service distribution contributes to the reduction in the equality. Moreover, recent advantages in geotechnology allow to accomplish more detailed measures that can contribute to maximize geographic access of the population (Guagliardo, 2004; Luo & Qui, 2009; Ribeiro et al., 2015; Zenk et al., 2005). For this fact, further research is needed to develop planning practices and policies that contribute to bringing together these inequalities and which may be used as decision support systems.

According to the Portuguese Health Ministry, the multidimensional nature of adequate access to health care results from the unavoidable link between geographic dimensions: availability and proximity (Furtado & Pereira, 2010). Proximity is one of the components that exceeds regarding access to health services (Law et al., 2011). It is directly related to the costs since it requires moving and rarely is taken into account.

The Portuguese Emergency medical services were reorganized in 2007, with the aim of achieving a better and more improved quality of emergency service, accessibility, and equity in regard to health (Ministério da Saúde, 2007). In this context, the National Health Plan 2012–1016 (extension to 2020) guidelines stressed efficiency and proximity needs as its main goals (da Saúde, 2012, 2015). However, 5 years on and very little research has been undergone to evaluate and acquire these initial goals. Moreover, the policy documents rarely take into account the geographical accessibility. Due to this inequitable geographic distribution of health‐care resources still in need of reorganization, this continues to be a major problem in Portugal.

If the provision of health care is a right of all citizens, then the coverage of health services should be provided for properly and according to the population's needs (Paez et al., 2010). Geographic Information System (GIS) represents information through layers and enables one to collect, organize, manipulate, and display geographic data in the aim of answering geographic questions (Remoaldo et al., 2017). It is also an important tool to identify patterns or design location scenarios that may be more suitable for health services. Furthermore, geoprocessing tools increase substantially in order to enhance a more complex and thorough database exploration (Ribeiro et al., 2015a).

Recent approaches to spatial analysis allow users to obtain more realistic and complex simulations/scenarios in finding more equitable solutions. Due to this, it is fundamental to explore the tools capabilities in regard to health planning. This research aims to contribute to this development, namely, to identify the levels of access by NUTS II and to apply the recent accessibility measure (E2SFCA) to the Mainland Portugal concerning the emergency services of the national health care. It can be distinguished from other accessibility measure studies by its integration of network data in spatial accessibility measures: the *enhanced two‐step floating catchment area* (E2SFCA). According to the implementation of this measure, it was found that accessibility levels have decreased, particularly for the most disadvantaged.

This paper aims to propose methodological enhancements in health‐care planning, measuring potential spatial accessibility to the Portuguese Emergency Services provision by improving the E2SFCA.

This paper has the following sections. Section 2 is dedicated to the literature review regarding health‐care accessibility and discusses the concepts of equity and accessibility, while section 3 is about methods and the data used in this research. Results are explored in section 4, and a discussion is provided in section 5.

## 2 Accessibility and Health Care

### 2.1 Equity and Accessibility

Hospitals are an important component of health‐care systems, and distance may still be an important barrier in reaching “health care for all.” Proximity plays an important role in access to health‐care service, and inequitable access to health care is a major problem for its planners (Mao & Nekorchuk, 2013), namely, when considering the inequalities that occur in health throughout Europe. Access to health care is a multidimensional concept, and it can be pointed out from two broad stages. The first one is the “potential” for the care delivered followed by the “received” one. The received one occurs when the population overcomes the provision barriers (Guagliardo, 2004). In fact, Penchansky and Thomas (1981) have grouped those barriers in two spatial dimensions (availability and accessibility) and three aspatial (affordability, acceptability, and accommodation) known as the five As. In this research, the spatial dimensions are measured. In fact, the availability refers to the services available for the population's use, while accessibility is a potential measure that highlights health‐care delivery (Guagliardo, 2004; Hawthorne & Kwan, 2013).

The actual Portuguese National Health Plan considers four dimensions: (i) citizenship in health, (ii) equity and adequate access to health care, (iii) health quality, and (iv) healthy policies (Ministério da Saúde, 2015). The health‐care access is one of the equity dimensions and that adequate access is one of the goals to extinguish access inequality. Nowadays, it is seen as a planned goal to promote spatial distribution in a balanced and fair spectrum, particularly when services delivered to the most vulnerable groups of social exclusion such as elderly are in cause (Ribeiro, Remoaldo, & Gutiérrez, 2015; Ribeiro, Remoaldo, Puebla, & Ribeiro, 2015; Yang et al., 2006). These are the main users of health services, and their economic conditions can amplify this disadvantage and promote the social exclusion phenomenon. Reducing distance can diminish travel time and cost in accessing health services while greater distances affect the probability of using the mentioned health services (Bissonnette et al., 2012; Higgs, 2009; Rosero‐Bixby, 2004).

Many times, authors, in accessibility analysis, look for time spent to reach health facilities. Time travel is analyzed as a proxy of health service proximity. Therefore, accessibility should be used as a measure that integrates travel distance and time between distinct locations (Guagliardo, 2004; Wong et al., 2012; Yang et al., 2006). Increasing barriers, such as distance to health, can stem users' ability to access services (Hiscock et al., 2008; *in* Bissonnette et al., 2012, Scholz, et al., 2015). Geographical accessibility can link two important components: (i) the volume of services provided in relation to the size of the population and (ii) the proximity of services in relation to geographical location (McGrail & Humphreys, 2014).

In the United Kingdom, health policies are driven by inclusion issues, and this system has been since the 1970s the baseline guide for the health Portuguese system. Indeed, the U.K. system was designed in 1948, and today, one of the key objectives of the political agenda for social inclusion lies in the need to provide equitable access to health services of the groups most vulnerable (Higgs, 2009). In the United Kingdom, accessibility is at the core of inclusion‐oriented policies, where transport plans have a necessity for accessibility planning and inclusion (Social Exclusion Unit, 2003). As a result, in the United Kingdom, some investigators are now trying to understand the levels of accessibility of the population in order to increase the accessibility of the population to health services (Langford & Higgs, 2006). Guagliardo (2004) stressed the need for studies that contribute to the understanding of barriers and taking into account land topography or the characteristics of road networks. In Portugal health‐care policies have been focusing on the assessment of accessibility on a national level bypassing higher geographical scales analysis or optimal health facility locations.

### 2.2 Accessibility Measures Used in Health‐Care Analysis—Assumption of Gravity Models

GIS are increasingly used to measure the impacts of geographic accessibility. Gradually, the network's capacity in GIS has favored the implementation of in‐depth geographic analysis and enabled the incorporation of other relevant elements such as the calculation of travel times and routes with shorter routes to reach health services (Higgs, 2009). Nowadays, for planning purposes, accessibility is widely recognized as a relevant indicator to integrate transportation and land use studies (Boisjoly & El‐Geneidy, 2016). Preston and Raje (2007) suggest an extra “matrix of area accessibility, area mobility and individual mobility” in a social exclusion/inclusion context.

Even though accessibility is a key concept in the study of transports and mobility, there is no consensus for a standard measure that should be adopted (Langford et al., 2012). *Distance measurements* have attracted several researchers to assess geographical accessibility (Fone et al., 2006; Jones et al., 2010; Sander et al., 2010; Wong et al., 2012). Traditionally proposed measures are simple and focus on distance measures or time travel between two points (Ingram, 1971), although one reference point affects several locations with distinct levels of accessibility. Beyond the distance measurements, there are four major approaches to assess accessibility: the *gravity‐based measures* (Geertman & Van Eck, 1995; Gutierrez & Gómez, 1999; Hansen, 1959; Schuurman et al., 2010), the *cumulative‐opportunity measures* (Wachs, 1973), the *space‐time measures* (Hägerstraand, 1970; Kwan, 1998; Miller, 1991; Weber & Kwan, 2002), and the *utility‐based measures* (Delafontaine et al., 2011; Dong et al., 2006; Small, 1992—Table 1).

**Table 1:** Main Approaches Used to Measure Accessibility

Models	Gravity‐basedmeasures	Cumulative‐opportunitymeasures	Space‐timemeasures	Utility‐basedmeasures
Description	▪ Measures of thegravitational type. ▪ Reach of the locationdue to attractiveness andcost of transportation.	▪ Opportunity‐basedmeasures. ▪ Obtaining opportunitiesavailable at a certain distance,travel time, or cost.	▪ Spatiotemporal measures. ▪ All the activitiesof the individuals have tobe inserted in a spatial andtemporal dimension. ▪ It measures the limitationof individuals.	▪ Measures based onthe advantages ofthe options. ▪ Treatment ofalternatives as randomvariables. ▪ Individual optionsdepending on themaximum usefulness.
Limitations	▪ Need to create animpedance factor. ▪ It considers the accessibilityof the place and not theindividual accessibility. ▪ Results are difficult tointerpret, based on measuresof accessibility defined asa potential indicatorof interaction.	▪ It does not considerthe impedance to reachcertain areas of supply,given that all opportunitiesare considered equal. ▪ Travel time or distanceis defined arbitrarily.	▪ Difficult applicationand operation. ▪ Need to ensure a highamount of data. ▪ There is no agreementbetween the results ofthese measures andthose carried outwith traditionallocalization measures.	▪ Complex theories, withdifficult interpretation. ▪ Difficult comparisonbetween utilitarianfunctions. ▪ Requires complexdatabases andcalculations.
Orientation	Attraction accessibility measures	Attraction accessibility measures	Constraints‐oriented approach	Benefit accessibility measures
Authors	(Geertman & VanEck, 1995; Gutierrez & García‐Palomares, 2007; Hansen, 1959).	(Wachs &Kumagai, 1973).	(Hägerstraand, 1970;Kwan, 1998; Miller, 1999;Weber & Kwan, 2002).	(Dong et al.,^2006^; Small, 1992).

Source: Own elaboration.

The gravity‐based method is the most used to model spatial interactions. It is employed to measure the force of attraction (*decay values*) for a kind of travel cost (Rodrigue et al., 2009). Thus, close locations have higher accessibility (Geertman & Van Eck, 1995; Hansen, 1959). The limitation of the gravity measure is that this method takes into account the supply but not the demand (Dong et al., 2006) despite the attempt of the latest researches try to include it (Wang & Tang, 2013). Gravity models are useful because they combine the quantity and/ or quality of health‐care facilities with the impedance of travel. Health‐care studies work with decay functions by parts for band widths defined a priori for travel distance. A simple way to evaluate the decay function is to admit the values of 0 and 1 as a function of the health services supply, considering that this measure of severity is of increasing opportunities, or known as proximity measurement.

*Cumulative‐opportunity measures* or isochrones are frequently recognized as easier to understand and interpret by the general public. It evaluates accessibility in terms of the number of opportunities that can be reached within a specified travel or time distance (Kwan, 1998) determining that a location with more opportunities is expected to have higher accessibility (El‐Geneidy et al., 2006). This approach has been used as a simplest way to evaluate equity in access to such facilities (Gutiérrez, 2001; Ribeiro, Remoaldo, & Gutiérrez, 2015; Ribeiro, Remoaldo, Puebla, & Ribeiro, 2015). For instance, this measure does not consider the attractiveness (facility size) or the difficulty in reaching it (Ben‐Akiva & Lerman, 1979; El‐Geneidy et al., 2006; El‐Geneidy et al., 2011).

Accessibility is understood as the potential of an individual to reach available opportunities (Boisjoly & El‐Geneidy, 2016; Preston & Raje, 2007). However, a broad number of factors can influence the easiness to reach a place. Major factors are related to transport, the individual, land use, and time components (Geurs & van Wee, 2004) that can contribute to construct several more complex accessibility measures. Due to this, several accessibility measures can derive to incorporate some of these components, resulting in more complex measures. At a regional planning level, frame location‐based measures are widely used by policy makers, despite it ignoring individual components (Boisjoly & El‐Geneidy, 2016). Gravity and cumulative‐opportunity methods are the mostly used regarding location‐based accessibility measures (Boisjoly & El‐Geneidy, 2016).

In the literature, accessibility is also differentiated between active or passive. The first one is more focused on the person's characteristics, while the second one is mainly related with the available opportunities. The person's accessibility measures the easiness in carrying out activities in certain locations, while the passive accessibility measures the easiness of reaching it by potential users (Cascetta et al., 2013; Kwan, 1998). It has been recognized that passive accessibility has been less investigated despite the growth in fields of application (Cascetta et al., 2013).

It is difficult to have an efficient accessibility measure that incorporates the multiplicity of factors which influence it. As a consequence, the gravity method has been widely used in health accessibility studies over time (Gutiérrez & García‐Palomares, 2008; Liu & Zhu, 2004; Miller, 1999; Ribeiro, Remoaldo, & Gutiérrez, 2015). Moreover, recent upgrades have been implemented with the *enhanced two‐step floating catchment area* (2SFCA; Polzin et al., 2014; Yang et al., 2006).

### 2.3 Enhanced Two‐Step Floating Catchment Area

Two broad categories can be used to classify health‐care accessibility: revealed and potential accessibility (Luo, 2004). It is difficult to measure the revealed accessibility due to the absence of updated health‐care service use, therefore not being revealed the spatial inequalities of patients' hospital health‐care accessibility. Hence, to measure potential geographical accessibility, the technique of 2SFCA is growing in the most recent health‐care studies, namely, to measure primary health‐care access (Langford et al., 2016). The unprecedented development of GIS makes it possible to analyze in a more realistic way the links between facilities and the population distribution to index the degree of facility in accessing health services. One can consider that it is now easier to integrate and measure interconnections of spatial and nonspatial factors.

One of the examples of a gravity model is the 2SFCA and was first proposed by Radke and Mu (2000). One of the advantages of 2SFCA is that results are easier and more intuitive to interpret. Despite recent improvements, particularly for smaller areas, to surpass the two major weakness, that is, distance decay and catchments size demonstrating that new improvements needed to be performed (McGrail, 2012). According to the author in large geographical regions, population and services are spread out and dispersed that distance decay cannot be negligible within a catchment. In the same way, catchments may still be “assumed to be the same size for all populations and for all services.” A more consistent improvement has been developed by the E2SFCA method (Langford et al., 2016; Luo, 2014; Luo & Qui, 2009). Critics of this method suggest that it overestimates the population's demand on service sites, and therefore, a three‐step floating catchment area method was proposed by Wan et al., 2012. This improvement was made to incorporate competition among health‐care services by assuming that the population demand in regard to a health‐care service is influenced by the availability of other nearby health‐care services (Luo, 2014).

## 3 Methods and Data

### 3.1 Geographical Context

The Portuguese Health System aims to promote people's access to health care and, on the other hand, to economic efficiency and a better use of the public resources framework (Amado & dos Santos, 2009; Ribeiro, Remoaldo, & Gutiérrez, 2015; Simões, 2004).

**Figure 1:**
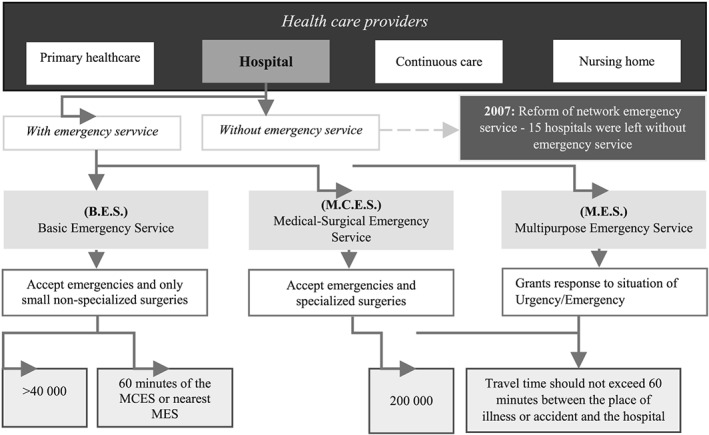
Structure of the Portuguese Emergency Service health‐care plan. Source: Own elaboration, based in Decree‐Law 725/2007

On November 2001, the Ministry of Health created the Portuguese Referral Network Emergency Hospital. Similarly, the Decree‐Law no. 157/99 of 07 February 2002 created the Basic Emergency Units and the Hospital Emergency Service. In 2007, the Ministerial Health Order number 17736/2006, of 31 August, proposed the upgrading of the Emergency Service and the operating area in the coordination of other emergency interventions. After that, the Ministerial Order 18459/2006 of 12 September defined three levels of response: Basic Emergency Service (B.E.S.), Medical‐Surgical Emergency Service (M.S.E.S.), and Multipurpose Emergency Service (M.E.S.). Nevertheless, the Order 727/2007 updates this network with the closure of 15 emergency services (Figure 1).

**Figure 2:**
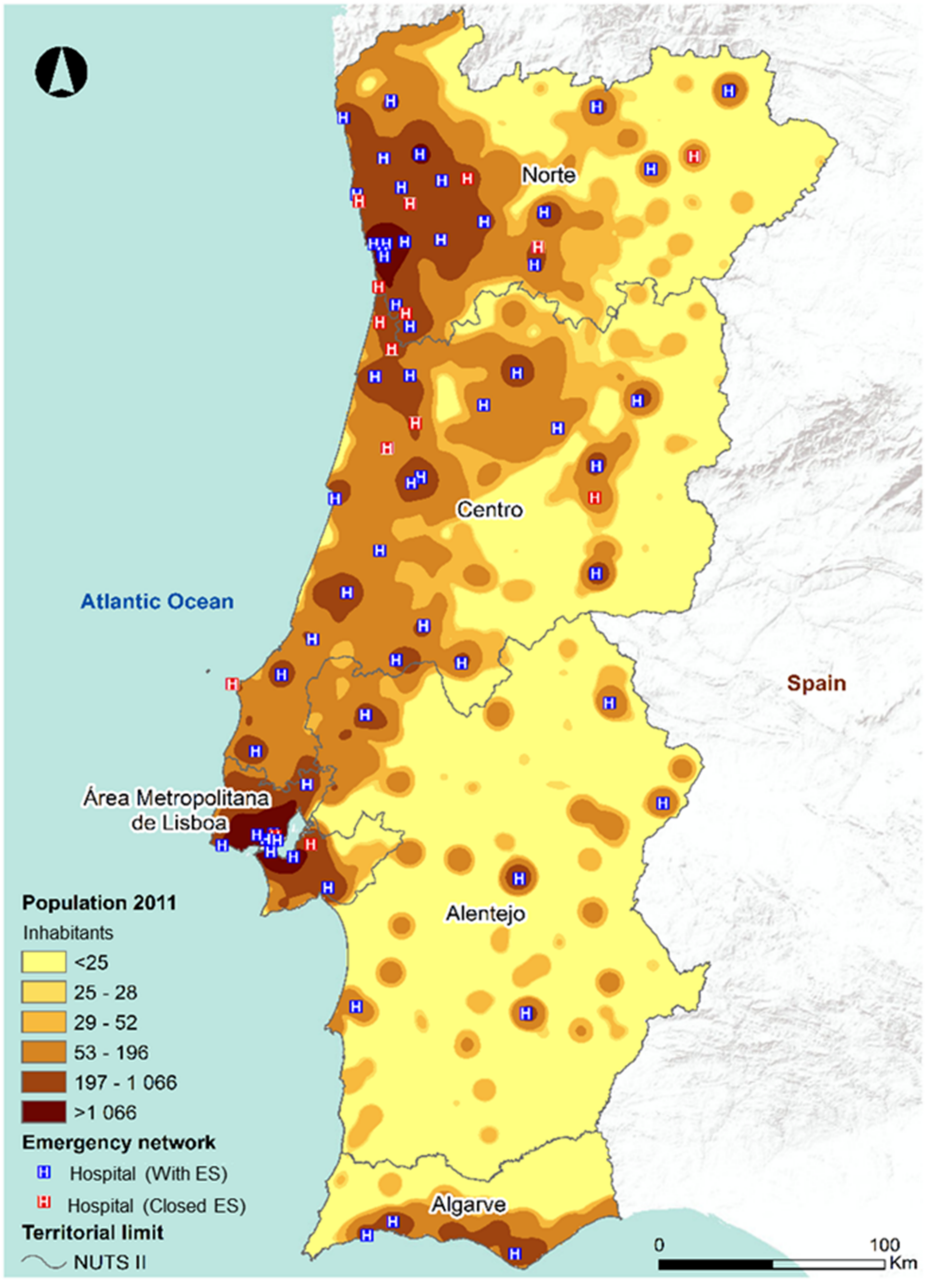
Emergency Service in health‐care providers' framework. Source: Own data, based in Decree‐Law 725/2007.

This reform caused the closure of 15 out of a total of 73 emergency services (E.S.) available before and aimed to improve the quality of treatment of urgent situations, proceeding to the rationalization of resources. In accordance with that order, several services and human resources that should underlie the hierarchical role defined for the emergency services (E.S.) have been articulated. In effect, those changes have resulted in decreasing the accessibility level in Portugal (Figure 2), particularly for the more vulnerable, like the elderly, young, and people that are physically dependent on others for medium‐ and long‐distance travel.

Emergency health‐care reform aimed to promote an equative distribution of facilities, privileging proximity in opposite to competition criteria (Polzin et al., 2014). Herein, we propose to improve the E2SFCA method, for the analysis of access to health care by adopting a methodology focusing on proximity analysis and including a distance decay. Emergency accessibility analysis was measured before and after the reform.

### 3.2 Methodological Procedures

Nowadays, there are a growing number of studies which seek to analyze the relationship between the levels of accessibility and the outcomes in health (Higgs et al., 2015). Even so, the number that interlinks the real speed and time and related cost is very low. As mentioned above, this study provides an evaluation of the improvements contributed by the Portuguese Referral Network Emergency Hospital and the main differences between the period before and after this reform.

For that purpose, we took a location model that required the organization of data into two groups: the location of Emergency Services (supply) and the centroid of population (demand). On one hand, we used the postcode for geocoding the public hospitals with Emergency Services. To each hospital the total physician's recorded by the Portuguese Regional Health Entity was associated. On the other hand, the 2001 and 2011 census data were used, opting preferentially for a maximum level of data disaggregation on a statistical subsection level. Although there are differences between the data structure of the statistical subsection in 2001 and 2011, the use of modeling for this purpose may lead to a more efficient and trustworthy analysis.

The study area includes Portugal's mainland territory with 58 hospitals with Emergency Services after the reform and 73 before it. The link between the supply and demand data was done by applying a network analysis to create an assessment focused on accessibility. We make use of the streets data supplied by ESRI Portugal. To measure accessibility a network analysis was used assuming a trip by ambulance which is normally used for emergency purposes, assuming a trip from the census track centroid. Congestion was not modeled. Catchments areas of 10, 20, and 30 min were also used as the distance decay function from equations ((1)), ((2)), and ((3)). Physician numbers were added to different time travel. In order to enhance the hospitals closest areas, a *distance decay* was used, defined by *β* = 1.15, and *Z* values of 1, 0.42, and 0.03 (different weights according to the Gaussian function (Luo & Qui, 2009). This function has a curve that varies depending on the distance. It seems to us the most realistic measure for more in‐depth and holistic analyses in fine‐scale studies).

(1) 1,if0<dkj≤Cj3or if0<dij≤ci32,ifCi3<dkj≤2Cj3or ifCi3<dij≤2ci33,if2Ci3<dkj≤cjor if2Ci3<dij≤Ci

(2) WkjorWij=1,ifdkjordij∈zone10.42,ifdkjordij∈zone20.03,ifdkjordij∈zone3.Ifβ=1.15.

For accessibility measure we opted for the formulation proposed by Wang and Luo (2005):

(3) $$\begin{array}{l} A_{i}F=\frac{\sum_{k\in \left\{d_{\mathrm{kj}}\in D_{r}\right\}}S_{j}\times W_{r}}{P_{k}}\\ \;=\frac{\sum_{k\in \left\{d_{\mathrm{kj}}\in D_{1}\right\}}{\mathrm{Sj}}_{1}\times W_{1}+\sum_{k\in \left\{d_{\mathrm{kj}}\in D_{2}\right\}}{\mathrm{Sj}}_{2}\times W_{2}+\sum_{k\in \left\{d_{\mathrm{kj}}\in D_{3}\right\}}{\mathrm{Sj}}_{3}\times W_{3}\;}{P_{k}}, \end{array}$$

where *A*_*i*_ *F* is the accessibility to the area *i*; *S*_*j*_ is the physician number available on the three time zones studied (*D*_1_, *D*_2_, and *D*_3_); *d*_*kj*_ is the time distance between *k* and *j*; and *d*_*r*_ is the distance time zone previously defined. *W*_*r*_ is the time distance accounted for the trip *r*th. *P*_*k i*_ is the population number located by statistical subsection. Accessibility index values were standardized according to the following formulation (4):

(4) $${\left(Z_{i}^{k}\right)}_{N}=\frac{Z_{i}^{k}-Z_{\min}^{k}}{Z_{\max}^{k}-Z_{\min}^{k}},$$

where $Z_{i}^{k}$ is the *Z* value at the statistical subsection; $Z_{\min}^{k}$ is the value of *Z* at the subsection that has the lower value; $Z_{\max}^{k}$ is the value of *Z* at the subsection that has the highest value. Because the values confined between 0 and 1 (a categorical variable) were seen as being the ideal situation with a value of 1.

This methodology allows us to answer to three questions about accessibility evaluation:

1. adequate accessibility levels are located at a *D*_*x*_ time distance limit of the influence area and values up that have less accessibility;
2. three distinct time distances were defined (*D*_1_, *D*_2_, and *D*_3_) with different impedance; and
3. emergency influence areas are defined by overlapping layers to count the available physicians at different Emergency Services.

## 4 Results

As is already a reference opportunity, a National Emergency Service Reform (S.N.S.) has brought about a few accessibility standards. For this reason, a significant part of the Portugal's mainland population, especially in the more deprived areas, was more exposed to the absence of an Emergency Services, within a 30‐min time span. Table 2 shows the population with an Emergency Services across time distance between the years 2001 and 2011.

**Table 2:** Levels of Accessibility to Public Emergency Services Between 2001 and 2011 in Mainland Portugal

Isochrone (min)	2001		2011		∆	
	Number	%	Number	%	Number	%
0–10	3,827,538	39.2	3,591,974	34.0	−235,564	−5.2
10–20	3,036,631	31.1	3,141,325	29.7	104,694	−1.4
20–30	1,596,692	16.3	1,709,454	16.2	112,762	−0.1
<30	8,460,861	86.6	8,442,753	79.9	−18,108	−6.7
>30	1,304,935	13.4	2,119,425	20.1	814,490	6.7

Source: Own elaboration.

The large differences in accessibility are intrinsic to the positive variation of the population that is more than 30 min away from the emergency facility. Between two analyzed decades, a decrease of 6.7% of inhabitants that lived 30 min away is apparent. In this regard, whereas in 2001, it was necessary to travel an average of 29 min to find an Emergency Services; the average time in 2011 was estimated in 41 min (or 12 min more compared to 2001).

**Figure 3:**
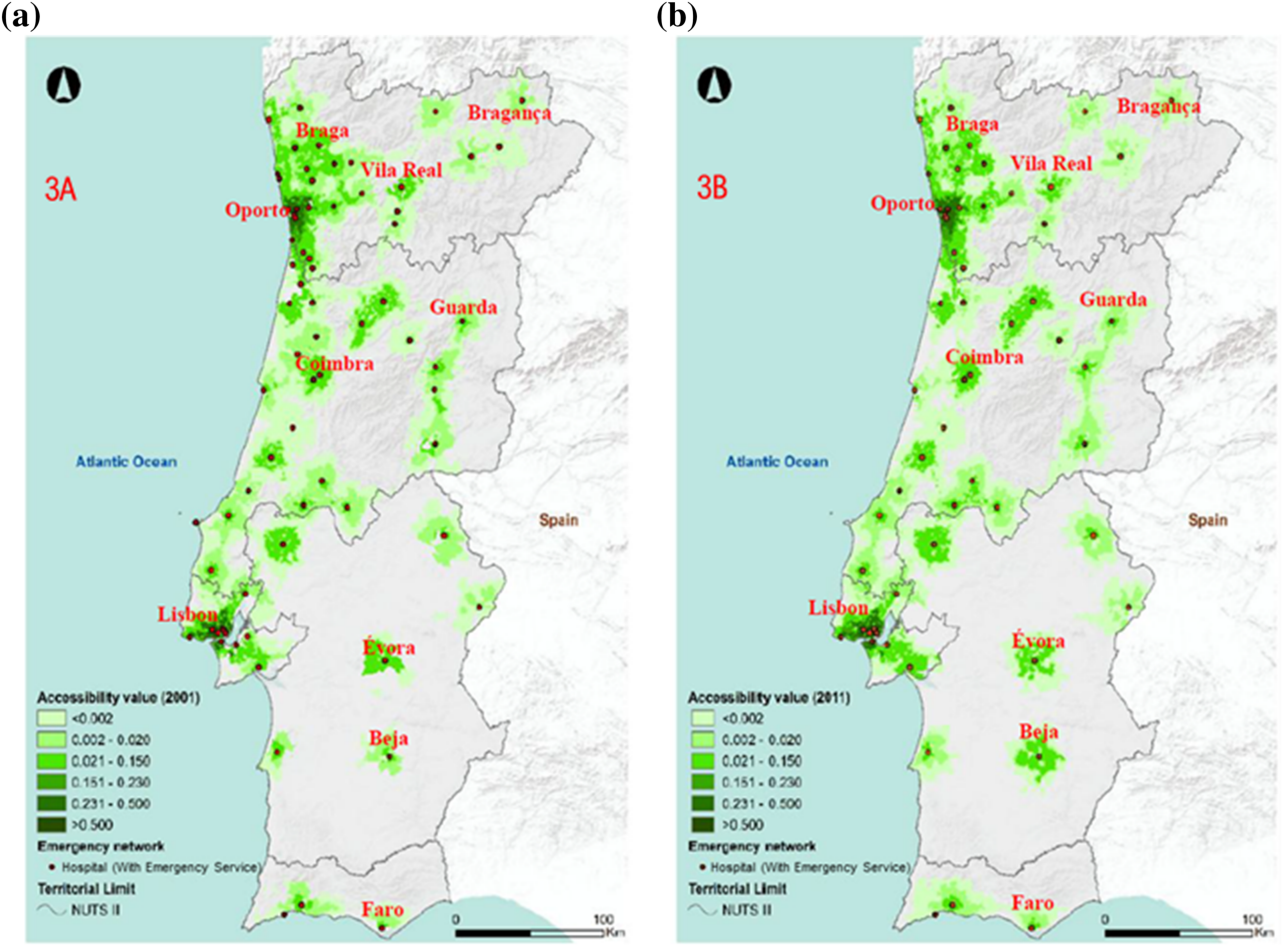
Levels of accessibility for (a) emergency service in 2001 and (b) emergency service in 2011. Source: Own elaboration

Figure 3 shows the results of the application of an accessibility measure using the 30‐min parameter for the acceptable time limit to access emergency services.

Thus, the lowest scores (close to 0) are in areas with more limited levels of access to health care, while the highest scores are in areas that the distance from an Emergency Services is minor. In both cases, there is a concentration of higher levels of accessibility in the coastal area of the Portuguese territory, due to the centralization of Emergency Services and, in many cases, the number of physicians available.

Regarding this, Luo and Whippo (2012)) stated that hospital services located in urban centers have a greater capacity to capture, as they influence the levels of accessibility in the surrounding rural areas. The figures recorded in the index confirm two assumptions: (i) mainland area registered a decrease in the average accessibility between 2001 and 2011 and (ii) there is a concentration of the highest average values, maximum and minimum accessibility in the NUTS II Metropolitan Lisbon Area (AML) and NUTS II North (Table 3), which are invariably the geographical areas with the highest population density and the lowest levels of deprivation of Emergency Services. In any event, it should be added that no attempt was made to classify Emergency Services by priority level. In any case, because the number of physicians is also used in the analysis performed, M.E.S. and M.C.E.S are known and have a greater need of physicians, whereas the B.E.S. is associated with a lower number of physicians.

**Table 3:** Descriptive Statistics of Accessibility Levels by NUTS II, in 2001 and 2011

Geographic location	2001			2011			2001–2011
	$\underline{x}$	Max	*σ*	$\underline{x}$	Max	*Σ*	$∆\;\underline{x}$
Alentejo	0.012	0.097	0.017	0.009	0.087	0.016	−0.003
Algarve	0.011	0.067	0.015	0.009	0.073	0.015	−0.002
Centro	0.011	0.241	0.035	0.010	0.282	0.022	−0.001
Área Metropolitana de Lisboa	0.090	1.000	0.174	0.083	1.000	0.166	−0.007
Norte	0.029	0.783	0.030	0.028	0.780	0.079	−0.001
Portugal Continental	0.022	1.000	0.066	0.020	1.000	0.065	−0.002

Source: Own elaboration.

It should also be noted that the levels of accessibility registered did not vary univocally in all the territories of mainland Portugal. All regions have decreased accessibility levels while the Lisbon Metropolitan Area continues to have higher levels of accessibility compared to other regions. However, with a loss of population with a score equal to or greater than 0.50 in the NUTS II Metropolitan Area of Lisbon. On the contrary, NUTS II North increased the total population in the 0.50–0.75 class, which may be due to a concentration of population still being present in the territories located to the west (e.g., a population increase, while those located in the inner fringe suffered a reduction—Table 4).

**Table 4:** Intervals Levels of Accessibility by NUTS II, in 2001 and 2011

Geographic location	2001								2011							
	0.00–0.25		0.25–0.50		0.50–0.75		0.75–1.0		0.00–0.25		0.25–0.50		0.50–0.75		0.75–1.0	
	Number (×10,000)	%	Number (×10,000)	%	Number (×10,000)	%	Number (×10,000)	%	Number (×10,000)	%	Number (×10,000)	%	Number (×10,000)	%	Number (×10,000)	%
NUTS II Alentejo	78	100	0	0	0	0	0	0	76	100	0	0	0	0	0	0
NUTS II Algarve	40	100	0	0	0	0	0	0	45	100	0	0	0	0	0	0
NUTS II Centro	235	100	0	0	0	0	0	0	233	100	0	0	0	0	0	0
NUTS II A.M. Lisboa	114	43	101	38	47	18	35	1	124	44	107	38	50	18	15	0
NUTS II Norte	300	81	61	17	81	2	0	0	296	80	587	16	14	4	0	0
NUTS I Portugal Continental	766	77	162	16	55	6	35	1	774	77	166	17	64	6	15	0

Source: Own elaboration.

## 5 Discussion and Conclusions

The results concerning the levels of accessibility to public emergency services between 2001 and 2011 show that a significant part of the territory is served by an Emergency Service at a tolerable distance time. However, accessibility levels show significant differences between urban and rural areas, namely, with the population of the two major urban centers (Lisbon Metropolitan Area and Porto Metropolitan Area). Indeed, the territories with higher levels of accessibility are associated with some of the municipalities in the center region, where the relationship between supply, demand, and distance time is evidently optimized.

2004 argue that population density alone corresponds to a relevant factor, since it can determine the demand for health care in each region. This methodological option offers several other options that allow the refining of index options within different weighting schemes placed in specific domains, depending on the context in which it may be applied, as in the study by Siegel et al. (2016).

It should be noted that this method of analysis can be useful to policy makers in shaping policies aimed at improving the distribution of public funding. From this analysis, the possibility of creating a more equitable access of the population stands out, with investments directed to the respective target populations. Nevertheless, the index can be remapped to other territories, although health systems may be considerably different in other countries. In addition, the inclusion of information regarding cross‐border health care in the analysis would improve the results of the Portuguese hospitals and populations located in border areas. Another of the analyses that could have been carried out was the use of more precise data relating to the hospital supply, namely, the obstetric and pediatric's services. In addition, as concluded in other studies (e.g., Langford et al., 2016), it is possible to choose this measure with a different viewpoint for different modes of transport, rather than focusing on only one mode of transport. On the other hand, recourse to measuring the distance between the place of storage of an emergency car and the centroid of the population to be served would be another improvement. Another useful evaluation, which was not the object of study, in this work is the evaluation of the accessibility to new emergency services, allowing to evaluate any existing flaws in the system.

In conclusive terms, the main changes to the emergency service led to a reduction in accessibility levels in rural areas, which was not the case in urban areas. Faced with this, the elderly population ended up being the most impaired. As it is very likely that in the near future will continue, in Portugal, to occur different regional access to health care, is needed to adopt policies at regional scale, and infrastructure and human resources planning, to decrease these differences. Health policies can still contribute to mitigate potential phenomena of social exclusion through the provision of localized emergency units of proximity, in order to address any levels of fragility that occur differently in Portuguese territory.

## Conflict of Interest

The authors declare no conflicts of interest relevant to this study.
